# Community responses to corona virus disease (COVID-19) in Africa in the face of “Infodemic”: A scoping review

**DOI:** 10.1016/j.parepi.2024.e00345

**Published:** 2024-02-27

**Authors:** Mikidadi Muhanga, Angela Jesse, Edwin Ngowi

**Affiliations:** Department of Development and Strategic Studies, College of Social Sciences & Humanities, Sokoine University of Agriculture, Tanzania

**Keywords:** Community responses, COVID-19, Myths, Misinformation, Misconception, COVID-19 “infodemic”

## Abstract

Globally, Corona Virus Disease **(**COVID-19) has significantly affected communities in various aspects. The World Health Organization proposed different measures to prevent the pandemic. However, these measures in some instances have not effectively minimized the impacts of COVID-19, due to innumerable factors, *inter alia,* considerable “infodemic” related to myths, misinformation, and misconceptions. Knowledge of the “infodemic” on COVID -19 can lead to effective interventions to rid societies of COVID-19, hence reduction of COVID-19-related risks and outcomes. This article explores the “COVID-19 infodemic” that affected community responses to COVID-19 in Africa. The study employed a scoping review approach involving peer-reviewed articles from numerous search engines and databases. The keywords involved in the search query were: “COVID-19 infodemic, COVID-19 false news, COVID-19 in Africa, ‘knowledge of COVID-19, ‘myths, misinformation, and misconceptions on COVID-19, ‘history of COVID-19’, ‘community responses to COVID-19 in Africa”. Findings show that 5G technology transferred coronavirus, high temperature and alcohol can kill coronavirus, blacks are immune to COVID-19, COVID-19 vaccine development has been rushed hence not very effective and safe and also causes infertility. Diverse community responses have been registered which in some ways frustrated efforts in combating the pandemic. Therefore, the “infodemic” consisting of myths, misconceptions, and misinformation have been resulting from the history of COVID-19 which first affected white people more than blacks. Also, low knowledge of how the virus is transmitted and affect human being; and the notion that COVID-19 affects richer than poor people, hence since white people are richer than black people then they were the first to be affected by the pandemic. Obviously in presence of such myths, misconceptions, and misinformation; community responses in combating COVID-19 have not been very effective in Africa. For these interventions to be effective, collective efforts involving various stakeholders to raise awareness of COVID-19 are needed.

## 1 Background information

Corona Virus Disease (COVID -19) is a pandemic that has created enormous attention worldwide (Saladino et al., 2020; Ausubiaro et al., 2021; Kesale et al., 2022; APS, 2020; Wang et al., 2020; Duan and Zhu, 2020; Garcia-Castrillo et al., 2020; Bartsch et al., 2020; Miethke-Morais, 2021). It was on 30th January 2020, the World Health Organization declared COVID-19 a Public Health Emergency of International Concern (PHEIC) (WHO, 2020). The pandemic was first discovered in China in 2019 and later spread to many developing and developed countries alike (Akat and Karataş, 2020; Balkhair, 2020; Khan et al., 2021; dos Santos, 2020; Al Mutair et al., 2022). The spread of COVID-19 connects very well to the free movement of people and pathogens, which is among the aspects of globalization. The WHO (2021) reported the first known and confirmed case of COVID-19 in Africa on 14 February 2020 in Egypt. Since then, global citizens have spent sleepless nights thinking of their future with noxious COVID-19 around.

It should be noted that this pandemic left no economic, social and political aspects uninterrupted (Oluyase, et al., 2021; Akat and Karataş, 2020; Burgess and Sievertsen, 2020; Cao et al., 2020; Kang et al., 2020; Ansell et al., 2021). COVID-19 is reported to have substantially affected the economies of countless countries (Bostan et al., 2020; Ngwakwe, 2020; Conte, 2020; Bartsch et al., 2020; Di Fusco et al., 2021; Gedik, 2020; Oluyase, et al., 2021; Kesale et al., 2022; Bilal et al., 2020) specifically on attempts to contain it. Wachyuni and Kusumaningrum (2020), uncover the incidences where COVID-19 limited global mobility involving tourists who normally do contribute to the income of various countries, African countries inclusive. Another effect aligns with human capital towards national development which connects to the loss of people's lives and particularly those who happen to stand as producers of goods and services. It is in the records that globally the total number of COVID -19 cases were 522 million while there were six million deaths attributable to the COVID-19 pandemic by the third week of May 2022. In Africa, COVID-19 cumulative cases of around nine million and 172,308 deaths have been reported since the region reported the first known and confirmed case of COVID-19 (WHO, 2022). The pandemic had also significantly increased the burden in terms of health services provision costs (Richards et al., 2022). It can be agreed to what dos Santos (2020) claims that losing dear ones through this pandemic has always had social and psychological implications, but also the literature (Kokou-Kpolou et al., 2020; Khosa-Nkatini and White, 2021) points out the manifestations along with the socio-economics of burial ceremonies, prolonged grief related to COVID-19 deaths and the up keeping of the would then be dependents if there will be any. Jain (2020) also posits that COVID-19 in terms of effects, on health-wise, has been associated with the development of acute coronary syndrome, congestive heart failure, myocarditis, and arrhythmias. The most valued cultural and traditional elements were seen to get into shambles, consider Africa, the home of handshaking and maybe congestion too, what a mess COVID-19 happened to be, leave alone other effects. It is very definite that COVID-19 through any lens really disturbed and interrupted the economic, social, and political aspects globally.

Understanding the socio-politiconomics of the pandemic was inevitable for governments and the community in the quest of creating innumerable measures to prevent the transmission of COVID-19. These measures included quarantine, total lockdown, insistence on the use of sanitizers, regular hand washing, use of face masks, physical social distancing, discouraging gatherings and visiting those suffering from COVID-19 (Nussbaumer-Streit et al., 2020; The Council of Economic Advisors, 2020; Brooks et al., 2020; WHO, 2020a, WHO, 2020b). On the same note, the WHO later, introduced different corona vaccinations to minimize risks resulting from COVID-19 including death (Jain, 2020; WHO, 2020a, WHO, 2020b).

The implementations of these measures in Africa were noted to have differed from one country to another depending on the social, economic even political contexts (U.S. Department of Labor-OSHA, 2020; WHO Regional Office for Africa, 2020; Pinchoff et al., 2021; Oyeyemi, 2021; Cahan, 2020; Godlee, 2020). For example, take the case of a lockdown, this was seen mostly being adopted by many developed countries in its totality but some developing countries were noted to adopt partial lockdown (Mboera et al., 2020; Ibrahim et al., 2020; Haider et al., 2020; Egger et al., 2020). Lockdown was among ways of maintaining physical social distancing which seemed only possible where the government concerned could afford the provision of support on basic needs to their citizens while in lockdown (Brodeur et al., 2021; Brown et al., 2020). However, considerable effects have been registered concerning lockdown. Since this measure denied an opportunity for those whose daily earning activities are not accomplished through some kind of online platforms hence that led to the reduction of household income. It should also be noted that the limited mobility emanating from the lockdown resulted in limiting individuals to attending to numerous social and political issues, *inter alia*, having fun; routine checkups for people with health problems and daily health care, political campaigns, burial ceremonies, prayers; leaving alone a lot of others (Arif et al., 2020; Atalan, 2020; Brown et al., 2020).

The literature indicates variations in terms of how the measures were perceived concerning minimizing the transmission of the COVID-19 pandemic in general. Some believed in their religious faith as a powerful weapon to fight COVID-19 rather than other proposed measures (Kowalczyk et al., 2020). Other studies also show the misconception and misinformation about COVID-19 from the media which created confusion for the community (Hossain et al., 2020). Moreover, different studies show that the myth, misinformation, and misconception of COVID-19 in terms of causes, transmission, and prevention were also observed in Africa hence leading to the spread of coronavirus and resulting in the loss of lives (Ahinkorah et al., 2020; Okereke et al., 2021; Omaka-Amari et al., 2020). It should however be noted that in some instances reports have not been presenting correct death records on COVID-19 (Ahinkorah et al., 2020). This went hand in hand with some acts in some countries either denying the existence of the disease or simply underreporting the incidences. Studies reveal that the myths, misconceptions, and misinformation about COVID-19 resulted from fear, worry and panic (Omaka-Amari et al., 2020). Thus, this review explored misinformation, myths, and misconceptions in African countries and how they affected the community response to prevent the transmission of COVID-19. Knowledge of misinformation, myths, and misconceptions about COVID -19 together with intentions and the ability to address them can lead to designing effective interventions towards the reduction of COVID-19-related risks and outcomes.

## 2 Methods

A scoping review method involving systematic search in numerous electronic bibliographic databases and search engines was employed to find relevant peer-reviewed studies. The protocol of the scoping review on misinformation, myths, and misconceptions about COVID-19 including the search strategy and the steps employed has followed similar protocols detailed in other studies including those by Audate et al. (2018); Levac et al. (2010); and Arksey and O'Malley (2005).

### 2.1 Data search strategy

The articles were identified from systematic searches of databases including AJOL, PubMed, CINAHL Plus with full text, MEDLINE (Embase), Academic search premier (EBSCO host), Google Scholar, CAB Abstract (ovid), and Web of science. A literature search was conducted from The English language original peer-reviewed articles published from 2019 were relevant to this review. The studies with original quantitative, qualitative, or mixed methods research were also involved. The other inclusion criterion was that studies should focus on COVID-19 in Africa and particularly on aspects of misinformation, myths, and misconceptions in connection to the community responses. Search queries involved keywords such as *“false news on COVID-19, COVID ‘infodemic’ ‘COVID-19 in Africa, ‘knowledge of COVID-19, ‘myths, misinformation, and misconceptions on COVID-19, ‘history of COVID-19’, ‘community responses to COVID-19 in Africa”*. Table 1 summarizes the inclusion and exclusion criteria for articles in the scoping review process.

**Table 1:** Inclusion and exclusion criteria for articles in the review.

Criteria	Included	Excluded	Justification for criteria application
Publication date	2019 to 2022	Before 2019	Used collections from carefully chosen databases to give a historical perspective on myths, misconceptions, & misinformation about COVID-19 in Africa
Publication language	Articles in the English language	Articles not written in English	To increase readability and due to co-authors' knowledge of English language only
Publication theme	Articles on myths, misconceptions, & misinformation about COVID-19 in Africa	Articles outside the scope of about COVID-19 in Africa	To remain within the scope of the scoping review
Availability of article	Fully available open-access articles	Complete articles not available	Due to not being open access, thereby requiring purchasing
Type of article	Peer-reviewed research journal articles	Conference abstracts, unavailable book chapters, review papers, bibliometric reviews and meta-analyses	Interested in available peer-reviewed empirical or original Research
Country or location of the study	Africa	None	To scope out the extent of myths, misconceptions, & misinformation about COVID-19 in Africa

### 2.2 Selection process

**Fig. 1:**
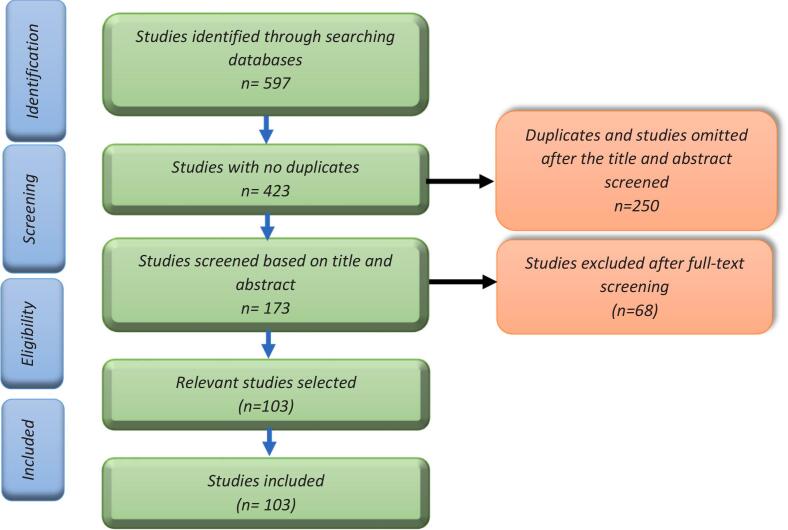
Articles selection process.

Three reviewers were involved (AJ, EN, MM) in screening the articles for selection in line with the eligibility criteria. The selection process began with the titles and abstracts screening, followed by a step that involved the screening of a full text. In case of incidences of any kind of conflicts emanating from the screening stages by the three reviewers, a discussion was initiated till consensus. Fig. 1 presents articles selection process.

### 2.3 Data extraction

Data extraction followed after the selection of the articles, this involved recording data such as author(s) name, year of publication, where published, study purposes and design (*e.g.*, mixed methods. Quantitative, or qualitative), and key findings. In this stage, while two authors were extracting the data, one author was involved in the validation of such data to ensure accuracy before the appraising of quality. Wallace et al., 2004 criteria and a modified rating system as suggested by Ohly et al., 2016 were employed in developing a checklist for the quality appraisal for the qualitative studies. This was followed by the preparation of a narrative account of the included studies to present patterns in misinformation, myths, and misconceptions. The outcomes were extensive, we had to synthesize them thematically to record the overall aspects related to misinformation, myths, and misconceptions.

Electronic databases searched brought 907 records (AJOL 187, Pubmed: 174, Embase: 191, Medline: 28, CINAHL Fulltext: 95, Academic search premier: 92, CAB abstract: 70, Web of Science: 70) that ended in 597 titles and abstracts which resulted from screening following the removal of the duplicates (Fig. 1). A total of 173 full-text articles were retrieved from various sources. The full-text screening stage led to 103 potential articles relevant to our scoping review. Additional articles were excluded after full-text assessment for various reasons. A total of 103 articles were therefore included in our final data extraction, quality appraisal, and narrative account stages.

## 3 Results

### 3.1 Included studies

This scoping review used standard systematic review methods to identify, select, and synthesize findings from 103 studies that reported on myths, misinformation, and misconceptions about COVID-19 in connection to community responses. The peer-reviewed literature on community responses to Corona Virus Disease (COVID-19) in Africa focusing on misinformation, myths, and misconceptions was analyzed thematically.

### 3.2 Data synthesis

Innumerable myths, misinformation, and misconceptions have been reported from the review. However, this review had inherent limitations concerning the fact that the review considered only English language bibliographic databases and journals; this may have disregarded other non-English which could have been relevant for this study. It should be borne in our mind that Africa is rich in terms of languages hence some myths, misinformation, and misconceptions could have not been identified and discussed as the review restricted itself to English language publications.

The origin, eruption, and prevention of COVID-19 had raised different misconceptions, misinformation, and myth that have been hindering the process of combating it. Many people have died and others suffered due to that. The reviewed studies present the myths, misconceptions, and misinformation about COVID-19 in Africa. The identified myths are summarized in Table 2 and discussed in the ensuing subsections.

**Table 2:** The reviewed studies on the myths, misconceptions, and misinformation of COVID-19 in Africa.

Author(s) and year	Geographic focus	Myths, Misconceptions, and Misinformation COVID-19
Lamptey et al. (2022)	Africa (various countries)	“researchers rushed the development of the COVID-19 vaccines; therefore, it is not very effective, safe and cannot be trusted” “After getting the COVID-19 vaccine, one can stop wearing a mask as well as taking safety precautions.”
Ovenseri-Ogbomo et al. (2020)	sub-Saharan Africa	COVID-19 is associated with the 5G technology
Kunguma (2021).	South Africa	The vaccine will cause infertility
Wirsiy et al. (2021)	Africa	COVID-19 vaccination affects the human body which leads to death The false conspiracy theory, rumors, and misinformation are the hindrances in accepting and responding to the COVID vaccine Religious myth on the COVID-19 & the vaccination
Broughton and Jarvis (2020), Hancocks (2020)		Vitamin D prevents and treats COVID-19
Okereke et al. (2021)	Rural Africa	Religious and political information was against the scientific facts (created misconception) COVID-19 cannot spread at extreme temperature The COVID-19 virus is not a reality its fake COVID-19 is the Government's avenue to secure more fund COVID-19 affects people with Higher Economic Status those with low economic status are immune to the virus Inhaling steam is highly effective against coronavirus The genetic makeup of African provides strong immunity against the virus
Osuagwu et al. (2021)	Sub Saharan Africa	COVID-19 was designed to reduce the population If you can hold your breath for 10 s you are not infected with coronavirus Drinking hot water flushes that virus COVID-19 has little effect on black than white people
Kabakama et al. (2022)	Cameroon, Ghana, Kenya, Liberia, Nigeria, Sierra Leone, South Africa, Tanzania, Uganda, and Zimbabwe.	sub-Saharan Africans were less vulnerable to COVID-19 a vaccine is not very effective, unsafe and should not be trusted
Aiyewumi and Okeke (2020)	Nigeria	Blacks are immune to COVID-19
Reuters (2020)		‘False claim: Steam therapy kills coronavirus’
Paget and Kwayu (2020)	Tanzania	Believed that COVID-19 cases were being artificially exaggerated and trusted herbs from Madagascar

### 3.3 Myths, misconceptions, and misinformation about COVID-19 in Africa

#### 3.3.1 5G technology and the spread of COVID-19

COVID-19 transmission was related to 5G technology as shown in Table 1. For example, the study by Ovenseri-Ogbomo et al. (2020) shows that some adults in sub-Saharan Africa believed that 5G technology is directly connected to the transmission of the COVID-19 pandemic. Other studies (Bruns et al., 2020; Mantica, 2021) reveal that the 5G rumor which was circulated through different social media has corrupted the minds of people in various countries.

#### 3.3.2 Temperature and the spread of COVID-19

It is indicated in Table 1 that there was different circulated information on media which stipulated that coronavirus cannot resist high temperatures, therefore; inhaling steam and exposing yourself to the high temperature can serve from contaminating coronavirus (Dutta et al., 2020; Izah et al., 2020; Le Page, 2020). Inhaling steam was practised by many people in both developing and developed countries, Mshana et al. (2021) report that in African countries, including Tanzania, people were boiling different herbs and inhale the steam as a way of curing and preventing the spread of coronavirus. Despite this belief, the debate is inconclusive among scientists with respect to the validity of the claims (Abraham et al., 2020; WHO, 2022).

#### 3.3.3 Whites *versus* blacks and COVID-19

Table 2 further shows that most Africans believed and are still believing that black people are immune to the COVID-19 pandemic (Osuagwu et al., 2021). The logic that connects to this belief rests on the observation that COVID-19 onset witnessed more white people infected and dying compared to Blacks. Other studies (Jean-Baptistea and Green, 2020; Laurencin and McClinton, 2020; Maqbool, 2020; Tirupathia et al., 2020) found that the myth that Africans are immune to COVID-19 has resulted in the loss of lives to more blacks than whites. Contrary to the myth, it is in the literature that, the coronavirus attacks every individual regardless of race (Laurencin and McClinton, 2020; Kendi, 2021; Saini et al., 2021).

Another misconception is that the pandemic is for richer people not for the poorer (Okereke et al., 2021). The said misconception had created a huge room for the transmission of the coronavirus to highly populated countries including Africa. It is hindering the acceptance of the directed measures which led to many deaths in African countries. The misconception was outsmarted by different studies which revealed that fighting against COVID-19 was harder for poor people than for richer ones (Maqbool, 2020; Laurencin and McClinton, 2020).

#### 3.3.4 Alcohol myth and COVID-19

Results in Table 2 show that alcohol was among the proposed medicine for COVID-19 during its outbreak where people decided to drink alcohol when they felt like having any signs of COVID-19 (Okereke et al., 2021). This misconception is built on the issue of using hand sanitiser with a certain percentage of alcohol. The misconception is cleared through literature which connotes that, alcohol is useful in preventing the transmission of the coronavirus specifically when a hand sanitiser contains about 60–95% of ethyl alcohol but, drinking it offers no COVID protection (National Institute on Alcohol Abuse and alcoholism (NIH), 2020; Neufeld et al., 2020; Prajapati et al., 2022). Different scholars have added that the use of alcohol harms human health because it affects the lungs which are accelerating low body immunity and makes an individual more vulnerable to COVID-19 (Andreasson et al., 2021; Bailey et al., 2021).

#### 3.3.5 Myths on vaccination

There has been a lack of reliable information which acted as a barrier to the uptake of COVID-19 vaccines (Mundagowa et al., 2021; Dinga et al., 2021). These myths include the claim that COVID-19 vaccination affects the human body which can lead led to death (Wirsiy et al., 2021). Lamptey et al. (2022) point out a claim that researchers hurried into COVID-19's vaccine development; consequently, the vaccines have been less effective, and unsafe and also cannot be relied on. On the same note, Kunguma (2021) reports that there is a widely held claim that vaccines can cause infertility.

## 4 Discussion

The identified 5G myth acted as a hindrance in combating COVID-19 since it led to the creation of negative perceptions towards social media especially when this claim could not be proved. The 5G technology was connected to COVID-19; it is the mass media that significantly propagated this.

There were information on media with respect to the failure of coronavirus to resist high temperatures, hence; people opted to inhaling steam and exposing themselves to the high temperature to avoid contaminating coronavirus. This resulted into this practice in various parts of developing and developed countries. This belief has been proved by scientists. However, it is obvious that too much steam inhalation can result in dry skin as well as damaging nose and throat cells hence affecting the body's immunity.

There has been a belief among some Africans that black people are immune to the COVID-19 pandemic. This belief connects to the observation that COVID-19 onset witnessed more white people infected and dying compared to Blacks. Contrary to the myth, the coronavirus has attacked every individual regardless of race. Ihe important issue is adhering to health guidelines regarding the pandemic and working on individuals' healthy life hence improving the body's immune system to fight against diseases. Similarly, there has been a misconception that the pandemic is for richer people not for the poorer. The said misconception had created a huge room for the transmission of the coronavirus to highly populated countries including Africa. This then proved that the claim was wrong as COVID-19 happened to affect both rich and poor people.

There was a misconception that alcohol could serve as medicine for COVID-19, this was particularly noted during its outbreak where people decided to drink alcohol when they felt like having any signs of COVID-19. This implies that people chose alcohol as a cure for COVID-19 out of ignorance. In the long run, the use of alcohol affected human health particularly the lungs.

There has been a lack of reliable information which acted as a barrier to the uptake of COVID-19 vaccines. These myths include the claim that COVID-19 vaccination affects the human body which can lead led to death and also cause infertility. This has led to hindrances in accepting and uptake of COVID vaccine. This connects very well to low vaccine uptake in Africa.

The overwhelming spread of information, particularly fake ones, amid the efforts in mitigating and preventing the pandemic has been seen to impede the pandemic response efforts (Gallotti et al., 2020). Generally, the myths have affected numerous interventions to contain the spread of the virus (Kunguma, 2021).

Some myths, including, that which claimed COVID-19 was transmitted through 5G, the myth stood as a blockage towards effective combating of COVID-19, as this claim could not be proven, it left a negative perception towards the social media in terms of its validity and reliability as a source of information. Social media could have been used to quickly disseminate COVID information hence educating communities and clearing myths, misconceptions, and misinformation.

They believe on inhaling steam to protect from the coronavirus (Dutta et al., 2020; Izah et al., 2020; Le Page, 2020; Mshana et al., 2021) has been observed to accelerate the infection within communities. Seemingly, came a point whereby people felt safer once had inhaled the steam, and this ended up opening doors wider for COVID-19 infection. It is in this context that, the provision of education on COVID-19 to the community on the spread and prevention remains imperative. Another myth that appeared to accelerate the spread of COVID-19 infection is based on the belief that white people were vulnerable relative to blacks. This belief in the long run resulted in relaxation in terms of adhering to the directives on preventing the spread of coronavirus to many Africans in developed and developing countries which led to the loss of their lives (Osuagwu et al., 2021; Jean-Baptistea and Green, 2020; Laurencin and McClinton, 2020).

Vaccines have been considered to have the potential in controlling the transmission of COVID-19. Myths about the COVID-19 vaccines have been reported to have resulted in low acceptance of vaccine uptake across Africa (Wirsiy et al. (2021); Kelen and Maragakis, 2021). Notably, it has been the concern of public health experts in Africa on low vaccination uptake, particularly in sub-Saharan Africa, as reported by WHO, 2020a, WHO, 2020b that there is a likelihood for some countries in Africa failing to meet the mid-2022 target of 70% vaccination rate.

At this juncture countries need to rethink their approaches to COVID-19, as has been pointed out in some literature (Jerving, 2021), a significant proportion of efforts should be directed at exploring the myths, misinformation, and misconceptions and ousting them and offering perfect information. These efforts are likely to encourage vaccination campaigns, and education, hence acceptance.

Some prospects vindicate the potential of social media in terms of collecting information and addressing rumors or popular beliefs. Currently, there are exponential incidences in terms of people who search for health information online. This is where the social media platforms are registering meaning concerning both the spread of dangerous COVID-19 misinformation and the accurate life-saving measures to be taken in the reduction of risk by communities, families, and individuals. This should be however taken with a cautious note that the same social media has had a negative influence with respect to “infodemic”. This calls for public health programs to have a proactive presence in information-sharing spaces but very warily provide access to information that is both reliable and correct.

Schmidt et al. (2020) opines that it is imperative to develop communication materials tailor-made-made for particular communities, hence assist lessening misconceptions related to COVID-19. Along with this, countries are advised to establish strategies that can significantly boost public understanding and limit misinformation around various responses in combating COVID-19, vaccines inclusive (Dzinamarira et al., 2021; Kunguma, 2021). These countries can learn from global responses meant to deal with infodemic. The major examples should be from the World Health Organization with events and programmes to combat fake news spreading including the third Virtual Global WHO Infodemic Management Conference, Africa Infodemic Response Alliance (United Nations, 2020) Early Artificial-Intelligence-supported Response with Social Listening (EARS)(World Health Organisation, 2021).

In an attempt to limit the ignorant population to consume fake news, an exemplary initiative in Africa comes from the government of South Africa which had to establish a National Coronavirus Command Council (NCCC). This council, *inter alia*, has been mandated with the task of advising the government and creating awareness of the pandemic. The government through the council went the extra mile. Declaring an attempt to share fake news is a criminal offence that constitutes a punishment through a fine or being jailed. There has also been established Digital Complaints Committee which is responsible for handling misinformation reports which are reported over a WhatsApp line or a hotline (Jimoh et al., 2020).

## 5 Conclusion and recommendations

It should be noted that there are enormous myths, misinformation, and misconception about COVID-19 inexistence in Africa. Undeniably, further understanding of the socio-politiconomics of the pandemic remains inevitable for governments and the community in the quest of creating innumerable effective measures to prevent transmission and contain COVID-19-related outcomes. There is an obvious indication that myths, misinformation, and misconception of COVID-19 identified accelerated transmission hence inhibiting combating of COVID-19 deaths in Africa. The most important is the fact a significant proportion of the population in Africa hesitated to take the vaccines based on misconstrued views that the vaccines could cause infertility. In absence of evidence, this remains a hard claim to advance. It will however remain in our mindsets, to quote Nelson Mandela that “education is the most powerful weapon which you can use to change the world”. This is an obvious call of duty to governments in Africa and beyond to create enormous knowledge on her population during this deadly pandemic, the assumption is that with some knowledge of the pandemic, some efforts can yield some extra results. Scientific research on COVID-19 is of great importance to prevent the destructiveness of COVID-19 in social, political, psychological, educational, and economic dimensions. After the COVID-19 epidemic has been controlled over time, the psychological effects on people will be clearer. It should be noted that education and awareness of pandemics cannot wait, it's time to clear these misinformation, myths, and misconceptions this has to begin with understanding them. In some ways a noteworthy proportion of the impacts of the pandemic would have been spared in presence of correct information on the pandemic, education as the case has always been will remain the number one job in curbing COVID-19. The need to emulate what has been attempted by the government of South Africa in the struggle to limit the infodemic remains very pertinent to minimizing the COVID-19 outcomes.

## Declaration of competing interest

The authors declare that they have no known competing financial interests or personal relationships that could have appeared to influence the work reported in this paper.
